# Chicago Society of Ophthalmology

**Published:** 1884-12

**Authors:** Boerne Bettman

**Affiliations:** Secretary


Chicago Society of Ophthalmology.

*June 9, 1884.*—The Society convened as usual, in the parlors of the Illinois Charitable Eye and Ear Infirmary. The following gentlemen were proposed for membership: Dr. Ira Marshall, Dr. Casselberry, Dr. W. H. Fitch.

Dr. Holmes made a verbal report of an interesting case, in which, two months after a very successful extraction of cataract, he divided extremely delicate capsular remains with a Knapp's needle-knife. The patient was greatly elated at the great improvement of her sight.

After a few days, the patient complained of irritation, although she had been confined to her room constantly, and to her bed four days. The eye had been under the influence of atropine and boracic acid. The conjunctiva, especially of the lids, was red. A small mass of mucus (?) was found on the edge of the lower lid and adherent to the globe. On the removal of this collection, it was observed that a minute filament extended from the needle puncture down over the cornea. There had been no pain. On the eighth day, vision had not suffered. More or less pain soon followed, with slight increase of tension. Eserin was substituted for the atropia. It was observed that a very fine, thread-like opacity extended into the vitreous. In a few days, the vitreous became more and more cloudy, the conjunctiva red and œdematous, and the eye painful. In a week more the patient was unable to count fingers.

The reporter was inclined to believe that the inflammation was not caused primarily by the injury of the intraocular tissue, but secondarily by the entrance of germs through the puncture from the minute prolapse of vitreous.*

At the conclusion of these remarks, Dr. Holmes invited the members to repair to the patient's room and examine the case at their leisure. The case was discussed at length. Owing to the great interest attached to it, Dr. Holmes was requested by a vote of the Society to prepare a paper on his experience in secondary operations for cataract.

Dr. G. Y. Gardiner read an exhaustive paper on "Boracic Acid in Ocular Therapeutics." The paper will soon appear at length. BOERNE BETTMAN, *Secretary.*

---

* We have since learned that the patient has greatly improved in all respects. Her sight, especially, is now quite good.

*August 12, 1884.*— Drs. Marshall, Casselberry and W. H. Fitch were elected to membership.

Dr. Hotz presented a pathological specimen with the following history: Mary S. (æt. 4) was first examined August 1, 1884. About ten days previously, without injury or any other manifest cause, the left eyeball became violently inflamed. She slept well, and never complained of pain. One or two days before her visit to the Dr.'s office, the patient was apparently sick; she had no appetite and no desire to play as usual. Before this inflammation set in, the left eye had the same appearance as the right. The following changes were noted by Dr. Hotz, August 1: Intense episcleral injection, interstitial haziness of cornea, retraction of iris, in consequence of which anterior chamber was abnormally deep. Pupil invisible, three-quarters of the anterior chamber being filled with a grayish-white substance. Tn. (?) fermentations were ordered. August 11, upper anterior portion of sclerotic discolored and distended (staphylomatous), injection of eyeball more intense, condition of cornea the same; anterior chamber filled entirely with the grayish-white substance.

The eyeball was enucleated and at once opened by a meridional section. Vitreous appeared wine colored and cloudy in its anterior half, the cloudiness being most dense near the origin of the ciliary processes and anterior limitans, which appeared densely white. Lens intact; pupil contracted; anterior chamber filled with a whitish-red vascular substance two mm. thick. It cannot be separated and distinguished from the iris tissue, and is firmly coherent with the posterior surface of the cornea. At the cornea, scleral margin, the anatomical boundaries of the various tissues are lost, all blend with the mass which has invaded cornea, sclera, and ciliary body. There is no trace of ciliary processes. The surface from ova serrata to pupil is perfectly flat and smooth. The posterior part of the eye has a normal appearance. The doctor expressed the opinion that the growth might prove to be a so-called granuloma on microscopic examination.

Dr. Boerne Bettman read a paper on "Amaurosis Following Hemorrhage." The two cases cited offered certain symptoms which, according to the doctor's opinion, pointed to a common cause, namely, to intra-cranial hemorrhage, with subsequent alterations of the optic nerve.

The first case, Margareth Feth, æt. 26, was admitted into the Eye Clinic of Heidelberg, 1879. She is of healthy parentage. Patient had repeated attacks of convulsions up to her fifth year; several brothers and sisters, seven in all, died a few months after birth from the effects of convulsions. Miss F. was a healthy, well-nourished child. Menstruation set in at the age of sixteen. On the 17th day of May, while at home, she suddenly experienced severe pain on the left side of her head, accompanied by dizziness and severe spells of vomiting. The pain increased during the night; a physician was hastily summoned; he prescribed powders, which apparently intensified the nausea and emesis. On the 21st, a second attack of sudden dizziness overcame her while in bed. She fell into an unconscious state, in which she remained twenty-four hours. While in this condition a large quantity of dark colored blood was seen by her mother to well from her mouth. On coming to, everything appeared dark about her. Investigation showed her to be perfectly blind. Sight returned to the right eye during the course of the day. A third attack of consciousness followed a few days later, leaving the sight of ther ight eye very much impaired. On the day of admission into the clinic there was complete amaurosis. Both pupils were dilated ad maximum. Irides did not respond to light. The ophthalmoscope revealed pronounced atrophy of both discs. Treatment of all varieties proved of no avail.

The second case was that of a boy, George Dohl, age three. The child fell against a chair, striking the upper teeth. The gums bled for three days, followed by severe convulsions, which lasted fully forty-eight hours. After the first twenty-four hours, during an interval of consciousness, the parents noticed that the child had lost sight in both eyes. Three days after the injury the child presented the following appearance: Pale, weak looking boy. Pupils dilated *ad maximum*, reaction to light prompt. Media both eyes clear. Papillæ very anæmic, almost perfectly white, caliber of arteries somewhat dilated. Amaurosis. The prognosis was unfavorable. Particular pains was taken to call the attention of the parents to one favorable feature of the case—the prompt reaction of the irides, which was interpreted as a sign of a partial restoration of vision. Sight gradually returned sufficiently to enable the child to roam about the house. Eight weeks after the accident, a second fit of convulsions ended fatally. The various theories of Graufe, Lammelsohn and Leber were then discussed.

Dr. Jefferson Bettman then read a paper entitled "Fracture of Base of Skull with Complication of Vago-Accessory nerves."

Mr. W., aged 45, consulted Dr. B. for hoarseness and deafness. Three weeks prior he had fallen off a wagon and sustained severe injuries on left side of head and face. This injury was followed by a sanguineous discharge from left ear, complete deafness on same ear, aphonia and aphagia. Intense headache, vertigo, vomiting and other cerebral symptoms were also present. A week later he was able to leave his room with but slight changes in the above mentioned main symptoms. At time of examination, gait was staggering, head was slightly drawn forwards and to the right side. Left *membrana tympani* showed traces of a recent rupture. Hearing power and bone conduction entirely gone on same ear. The posterior superior wall of external auditory meatus reddened and acutely sensitive to touch of probe. A constant *tinnitus aurium* caused the patient great annoyance. Further examination showed motor paralysis of left fauces and pharynx and almost complete anæsthesia of these parts. The left vocal cord occupied cadavernic position with no overlapping of the arytænoids. Palpation with a laryngeal probe, revealed a state of anæsthesia of the entire left larynx. Auscultation over the œsophagus yielded a distinct rumbling sound on attempted deglutition (*deglutitio sonora*). The left side of neck and head was painful on deep pressure. The patient was lost sight of and only after an interval of nine months did he again afford the observer an opportunity to examine the state of affairs. Sensibility was now restored to larynx and pharynx, but the left vocal cord was still in a paralytic state. The condition of of the left ear was in no way altered, the *tinnitus* being distressing. He still complained of dizziness aggravated on lowering his head and a constant dull headache. Large doses of iodide and faradization had no material effect in reducing these disturbances. The essayist then dwelt at great length upon the anatomy and physiology of the nerves affected, their relation to each other and their respective functions, innervation of the larynx, pharynx, etc. The present paper was merely a preliminary communication, further discussion and elaboration being reserved for future publication.

Dr. Holmes made a verbal report of an unusual case of upward strabismus. A boy 13 years of age had suffered from very early childhood from a peculiar turn of the right eye upward, which occurred about every half minute. There was not a quick spasmodic motion as in chorea but a slow movement as in certain cases of convergent strabismus. During the contraction of the *levator superioris* more than three quarters of the cornea was concealed under the upper lid the vision with—$\frac{1}{48}$ cyl. 90° was normal. Neither by the use of prisms or any other means could diplopia be induced. A division of the superior rectus almost absolutely relieved the annoying action of the muscle. At the end of 3 months the patient reported no return of the anomaly.

BOERNE BETTMAN, *Secretary.*

